# The intracellular bacterium *Rickettsia rickettsii* exerts an inhibitory effect on the apoptosis of tick cells

**DOI:** 10.1186/s13071-020-04477-5

**Published:** 2020-12-01

**Authors:** Larissa Almeida Martins, Giuseppe Palmisano, Mauro Cortez, Rebeca Kawahara, José Mario de Freitas Balanco, André Fujita, Beatriz Iglesias Alonso, Darci Moraes Barros-Battesti, Gloria Regina Cardoso Braz, Lucas Tirloni, Eliane Esteves, Sirlei Daffre, Andréa Cristina Fogaça

**Affiliations:** 1 Department of Parasitology, Institute of Biomedical Sciences, University of São Paulo, São Paulo, SP Brazil; 2 Department of Computational Science, Institute of Mathematics and Statistics, University of São Paulo, São Paulo, SP Brazil; 3 Department of Veterinary Pathology, São Paulo State University, Jaboticabal, SP Brazil; 4 Department of Biochemistry, Institute of Chemistry, Federal University of Rio de Janeiro, Rio de Janeiro, RJ Brazil; 5 Rocky Mountain Laboratories, National Institutes of Health, National Institute of Allergy and Infectious Diseases, Hamilton, MT USA; 6 Present Address: Rocky Mountain Laboratories, National Institutes of Health, National Institute of Allergy and Infectious Diseases, Hamilton, USA; 7 Present Address: Department of Molecular Sciences, Macquarie University, Sydney, NSW Australia

**Keywords:** Apoptosis, Proteome, Rickettsiae, Tick

## Abstract

**Background:**

*Rickettsia rickettsii* is a tick-borne obligate intracellular bacterium that causes Rocky Mountain spotted fever, a life-threatening illness. To obtain an insight into the vector–pathogen interactions, we assessed the effects of infection with *R. rickettsii* on the proteome cells of the tick embryonic cell line BME26.

**Methods:**

The proteome of BME26 cells was determined by label-free high-performance liquid chromatography coupled with tandem mass spectrometry analysis. Also evaluated were the effects of infection on the activity of caspase-3, assessed by the hydrolysis of a synthetic fluorogenic substrate in enzymatic assays, and on the exposition of phosphatidyserine, evaluated by live-cell fluorescence microscopy after labeling with annexin-V. Finally, the effects of activation or inhibition of caspase-3 activity on the growth of *R. rickettsii* in BME26 cells was determined.

**Results:**

Tick proteins of different functional classes were modulated in a time-dependent manner by *R. rickettsii* infection. Regarding proteins involved in apoptosis, certain negative regulators were downregulated at the initial phase of the infection (6 h) but upregulated in the middle of the exponential phase of the bacterial growth (48 h). Microorganisms are known to be able to inhibit apoptosis of the host cell to ensure their survival and proliferation. We therefore evaluated the effects of infection on classic features of apoptotic cells and observed DNA fragmentation exclusively in noninfected cells. Moreover, both caspase-3 activity and phosphatidylserine exposition were lower in infected than in noninfected cells. Importantly, while the activation of caspase-3 exerted a detrimental effect on rickettsial proliferation, its inhibition increased bacterial growth.

**Conclusions:**

Taken together, these results show that *R. rickettsii* modulates the proteome and exerts an inhibitory effect on apoptosis in tick cellsthat seems to be important to ensure cell colonization.
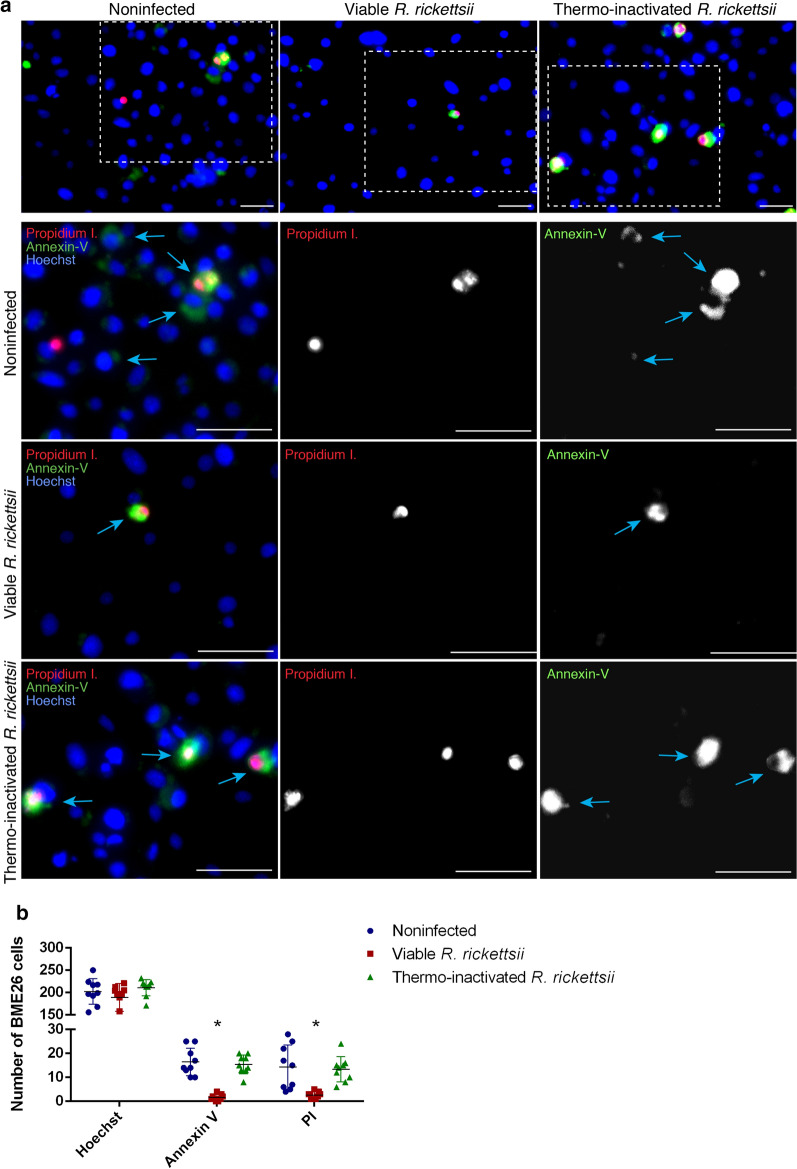

## Background

Ticks are hematophagous arthropods that parasitize almost all classes of vertebrates throughout the world, resulting in the transmission of a vast list of pathogens that threaten both human and animal health [1–3]. Among tick-borne diseases, Rocky Mountain spotted fever, which is caused by the obligate intracellular bacterium *Rickettsia rickettsii*, is one of the most severe [4–6].

The development of in vitro cell culture systems, particularly of continuous cell lines derived from tick embryos, has contributed significantly to the study of the interactions between ticks and tick-borne pathogens [7]. Of these tick cell lines, cell line BME26, which is derived from *Rhipicephalus microplus* embryonic cells, has been previously characterized by our research group [8]. Interestingly, these cells present phagocytic activity and constitutively transcribe different immunity genes, such as ferritin, heat-shock proteins, reactive oxygen species, antioxidant proteins, protease inhibitors and the antimicrobial peptides microplusin, defensin and ixodidin [8]. BME26 cells have been shown to be susceptible to *Borrelia burgdorferi* [9], the causative agent of Lyme disease, and to *Anaplasma marginale* [10], the causative agent of bovine anaplasmosis. This cell line has also previously been used as a model to analyze the transcriptional response upon exposure to *A. marginale* [11–13] and *R. rickettsii* [12, 13].

In the study reported here, we determined the proteome of BME26 cells in response to experimental infection with *R. rickettsii* using label-free liquid chromatography coupled with tandem mass spectrometry (LC–MS/MS). Interestingly, proteins of different functional classes were modulated in a time-dependent manner. Among the proteins related to apoptosis, certain negative regulators were downregulated at the beginning of infection (6 h) and upregulated at a later time point (48 h). Previous studies have also demonstrated that *R. rickettsii* is capable of inhibiting the apoptosis in human endothelial cells [14–16]; therefore, we evaluated the effects of *R. rickettsii* infection on DNA fragmentation, tick caspase-3 activity and phosphatidylserine externalization of BME26 cells. We also assessed whether the activation and inhibition of caspase-3 exerted an effect on bacterial proliferation. Taken together, our results show that *R. rickettsii* infection inhibits apoptosis in tick cells, which seems to be important to ensure colonization.

## Methods

### Cell cultures and *R. rickettsii*

The embryonic cell line of the tick *Rhipicephalus microplus* (Canestrini), BME26 [8], was cultured in L-15B300 medium containing 100 U/ml of penicillin and 100 μg/ml of streptomycin and supplemented as previously described [8, 17]. The embryonic cell line of the tick *Amblyomma sculptum* (Berlese), IBU/ASE-16 [18], was also cultured in L-15B300 medium supplemented with 2 mM l-glutamine. BME26 cells were incubated at 34 °C and IBU/ASE-16 cells at 30 °C. The number of viable cells per millilter was determined using an automatic cell counter (model TC20®; BioRad, Hercules, CA, USA) after staining with 0.4% Trypan blue solution. The genomic DNA (gDNA) extracted from both cell lines was periodically evaluated by PCR using specific primers for the 16S rRNA (forward: 5′-GGG AGC AAA CAG GAT TAG ATA CCC T-3′; reverse: 5′-TGC ACC ATC TGT CAC TCT GTT AAC CTC-3′) to confirm the absence of *Mycoplasma* spp. [19].

The highly virulent Taiaçu strain of *R. rickettsii* [20] was used in the experiments. First, an inoculum of *R. rickettsii* in tick cells (BME26) was obtained. To that end, *R. rickettsii*-infected Vero cells [21] were disrupted by heat shock (three cycles of incubation at 37 °C followed by 3 s in liquid N_2_) to release rickettsiae. The resulting cell lysate was transferred to BME26 cells. After 72 h at 34 °C, the medium was removed, BME26 cells were disrupted by heat shock, as described above, and the resulting cell lysate was used to infect additional BME26 cells. After 48 h at 34 °C, the medium was removed, the cells were harvested, suspended in supplemented L-15B300 medium containing 10% dimethyl sulfoxide (DMSO) and 5% fetal bovine serum and stored in liquid N_2_ before use. The same inoculum batch was used in all the experiments to guarantee equal infection. An aliquot of the infected BME26 cells was used for gDNA extraction and quantification of rickettsiae in the inoculum, as described below.

For all experiments, the culture medium was replaced by L-15B300 medium without antibiotics 3 days before infection. Cells were infected using a MOI (multiplicity of infection) of 10. As a control, cells were incubated with a lysate of noninfected BME26 or IBU/ASE-16 cells, disrupted by three cycles of incubation at 37 °C followed by 3 s in liquid N_2_.

 All experiments were conducted according to the recommendations of the Ministry of Health of Brazil.

### gDNA extraction

For gDNA extraction, cells were harvested and centrifuged at 3500 rpm for 10 min at 4 °C. The cell pellet was resuspended in 50 μL of RNA*later*® (Thermo Fisher Scientific, Waltham, MA, USA) and maintained at − 20 °C until use. The gDNA was extracted using the BlackPREP Tick DNA/RNA Kit (Analytik Jena AG, Jena, Germany) according to the manufacturer's specifications. At the end of the procedure, the gDNA was quantified in a spectrophotometer (model NanoDrop-1000; Thermo Fisher Scientific) and maintained at − 20 °C.

### Quantification of *R. rickettsii* by quantitative PCR

To determine the total number of rickettsiae in tick cells, the gDNA was used as a template in a quantitative PCR (qPCR) with specific primers and a hydrolysis probe [22] for the single copy gene *gltA* (citrate synthase) of *Rickettsia* spp. [23]. The gDNA extracted from noninfected cells (control) was also assayed to confirm the absence of infection.

### Protein extraction and digestion

Five biological replicates of BME26 cells infected with *R. rickettsii* for either 6 or 48 h were obtained. As a control, five biological replicates of BME26 cells incubated with a lysate of noninfected BME26 cells for 6 or 48 h were also obtained. After treatment, the cells were detached from the flasks with a cell scraper device into a sterile SPG buffer [24] containing a cocktail of protease inhibitors (P2714; Sigma-Aldrich, St. Louis, MO, USA). After centrifugation at 3500 rpm for 10 min, the cells were suspended in a lysis buffer (100 mM NH_4_HCO_3_, 8 M urea) containing the same cocktail of protease inhibitors described above, and then disrupted by thermal shock (three cycles of 2 min at 30 °C and 30 s in liquid N_2_). The protein concentration was determined using the bicinchoninic acid kit (BCA; Pierce™, Thermo Fisher Scientific) according to the manufacturer's instructions. From each biological replicate, 50 μg of proteins was reduced with dithiothreitol (DTT, final concentration 5 mM), alkylated with iodoacetamide (final concentration 10 mM) and digested with trypsin using a ratio of 1:50 (μg trypsin/μg protein) in 50 mM ammonium bicarbonate solution at 37 °C overnight [25]. The reaction was stopped with 1% formic acid, and 10 μg of the resulting peptides was desalted with ZipTip C_18_ (Millipore Corp., Merck Millipore, Burlington, MA, USA) and concentrated in a vacuum centrifuge.

### High-performance LC–MS/MS and data analysis

The desalting peptides were suspended in 0.1% formic acid and submitted to LC-MS/MS analysis. The peptides were separated on an EASY-Column (10 cm × 75 μm, 3 μm) analytical column coupled with a high-performance liquid chromatography (HPLC) Easy Nano-LC system (Thermo Fisher Scientific). For elution of the peptides, a linear gradient of 0 to 35% acetonitrile in 0.1% formic acid was used (75 min; flow rate 300 nl/min). The voltage of the nanoelectrospray was 1.7 kV and the temperature was 275 °C. The mass spectrometer (model LTQ Orbitrap Velos; Thermo Fisher Scientific) was operated in data-dependent mode, automatically switching between MS and MS/MS modes. MS spectra were acquired between 400 and 2000* m*/*z* on the Orbitrap analyzer, with a resolution of 60,000. The 20 most intense ions of the MS spectra were selected and fragmented by collision-induced dissociation with 35% normalized collision energy. All biological replicates were analyzed in duplicate.

LC-MS/MS raw files were imported into the MaxQuant version 1.5.2.8 program [26], and the search tool Andromeda [27] was used to search peptides against a database. This database (Additional file 1: FASTA file) was composed of deduced amino acid sequences from transcripts obtained by RNA-seq analysis of BME26 cells [raw data were deposited to the Sequence Read Archives (SRA) of the NCBI under bioproject number PRJNA607772, and the Transcriptome Shotgun Assembly (TSA) project has been deposited at DDBJ/EMBL/GenBank under the accession GINU00000000 (the version described here is the first version, GINU01000000)] and of protein sequences from bacteria of the genus *Rickettsia* available in Uniprot database [28]. For protein identification, a tolerance of 10 ppm was used for the precursor ion and 0.5 Da for the fragment ions. Parameters included: cleavage by trypsin with a maximum of two missed cleavages, carbamidomethylation of cysteine (57.021 Da) as a fixed modification, and oxidation of methionine (15.994 Da) as a variable modification. Peptide‐spectrum matches and peptides and protein false recovery rates (FDR) were kept below 1%. Only proteins identified from at least two distinct peptides were accepted. Protein quantification was performed using the LFQ (label-free quantification) feature in the quantitative proteomics software package MaxQuant. The differential proteome from infected cells in relation to control cells was determined using Perseus v.1.5.2.6 software. Only proteins with *P* < 0.05 (Student's t-test) with a multiple test correction (FDR) < 5% and with a relative abundance (fold-change) ≥ 1.5 or ≤ 0.67 in infected cells in relation to control cells were considered to be modulated. Proteins significantly different in relative abundance between infected cells and control cells but presenting a fold-change of < 1.5 and > 0.67 were considered to be unmodulated. Proteins identified exclusively in infected or control samples were also considered to be modulated (up or downregulated, respectively). The MS raw files were submitted to PRIDE (https://www.ebi.ac.uk/pride/) with the submission number PXD017942. The principal component analysis was performed using the online platform MetaboAnalyst 3.0 [29].

### gDNA fragmentation evaluation by agarose gel electrophoresis

The gDNA extracted from noninfected or *R. rickettsii-*infected BME26 cells at 6, 24, 48, 72, 96 and 120 h after the beginning of the experiment was analyzed on a 1% agarose gel electrophoresis stained with RED™ Gel (Uniscience Corp., Miami Lakes, FL, USA). After electrophoresis with a constant voltage of 100 V for 60 min, the gel was visualized under ultraviolet (UV) light in a transilluminator coupled to an imaging system (ImageQuant™ 300 model; GE Healthcare, Chicago, IL, USA).

### Enzymatic assays for evaluation of caspase 3 activity

The first step in the analysis of caspase-3 activity in BME26 cells, pre-infected or not with *R. rickettsii* for 24, 36 or 48 h, was to induce caspase-3 activity with staurosporine (final concentration 400 nM; Sigma-Aldrich). After an additional 6 h of incubation, cells were detached and centrifuged at 3500 rpm for 10 min at 4 °C. The cell pellet was then suspended in 80 μl of lysis buffer [20 mM piperazine-*N*,*N*′-bis(2-ethanesulfonic acid) (PIPES), 100 mM NaCl, 2 mM ethylenediamine tetraacetic acid (EDTA), 0.1% 3-[(3-cholamidopropyl)dimethylammonio]-1-propanesulfonate hydrate (CHAPS), 10% sucrose, 0.1% Triton X-100, 1 mM phenylmethanesulfonyl fluoride (PMSF), 2 μM pepstatin, pH 7.2], followed by centrifugation at 13,000 rpm for 5 min at 4 °C. The supernatant (cytosol) was collected and the protein concentration determined using the BCA kit (Pierce™, Thermo Fisher Scientific), according to the manufacturer's instructions. Aliquots of 50 μg of proteins from each sample were incubated at 37 °C with 50 μM of the Ac-DEVD-AMC synthetic substrate (Calbiochem®; Merck KGaA, Darmstadt, Germany) (FluoroNunc™ well plates; Thermo Fisher Scientific) in 50 mM HEPES (*N*-2-hydroxyethylpiperazine-*N*′-2 ethanesulfonic acid) buffer, pH 7.0, containing 10 mM DTT. The release of aminomethyl coumarin (AMC) fluorescence was continuously monitored for 1 h at 5-min intervals using a fluorimeter (SpectraMax® i13; Molecular Devices, San Jose, CA, USA) set at wavelengths of 380 nm for excitation and 460 nm for emission. The relative activity of caspase-3 was calculated using the ratio of the ΔUAF (units of arbitrary fluorescence; UAF_60 min_ − UAF_0 min_) of the target condition by the ΔUAF of the reference condition (ΔUAF_target condition_/ΔUAF_reference condition_). The same procedure was used to evaluate caspase-3 activity in BME26 or IBU/ASE-16 cells infected with *R. rickettsii* or challenged with heat-inactivated *R. rickettsii*. The inactivation of rickettsiae was obtained by exposure to heat at 56 °C for 1 h. After inactivation, rickettsiae were inoculated to BME26 cells and bacterial growth was evaluated after 6, 24, 48 and 72 h by qPCR. No bacterial growth was detected, showing that bacteria were efficiently inactivated by heat (data not shown). Forty-eight hours after exposure to rickettsiae, staurosporine (final concentration 400 nM) was added to the cells and caspase-3 activity was assessed as described above. Caspase-3 activity was also evaluated in uninfected cells as well as in cells nontreated with staurosporine, as a control. At least three biological replicates of each condition were analyzed in three technical replicates. Data were statistically analyzed by Student’s t-test using GraphPad Prism (v6.0; GraphPad Software Inc., San Diego, CA, USA), and the activity of caspase-3 was considered to be significantly different at *P* < 0.05.

### Evaluation of apoptosis by fluorescence microscopy

BME26 cells were cultured in a 35-mm Petri dish containing a 1.5 coverglass (Nunc™ Glass Bottom Dishes; 1.2 × 10^6^ cells per dish; Thermo Fisher Scientific) and maintained at 34 °C for 24 h. The BME26 cells were infected with *R. rickettsii* or challenged with heat-inactivated *R. rickettsii*; noninfected cells were used as the control. After 48 h, staurosporine (final concentration 400 nM) was added to the cells and the cells incubated for an additional 6 h, following which the cells were labeled for 15 min with 1 μl/ml of annexin-V conjugated to Alexa Fluor™ 488 (Invitrogen, Carlsbad, CA, USA), 0.25 μg/ml of Hoechst 33342 and 1 μl/ml propidium iodide (PI). The cells were then observed under a fluorescence microscope (model DMI6000B/AF6000; Leica Microsystems GmbH, Wetzlar, Germany) coupled to a digital camera system (model DFC365FX; Leica Microsystems GmbH). Three independent images obtained from three biological replicates were acquired and processed by the Leica Suite X (LAS X) software. The number of cells labeled with annexin-V was determined using Image J software [30]. The number of annexin-V-labeled cells for each treatment were analyzed using a Student’s t-test using GraphPad Prism software, and differences were considered to be significant when *P* < 0.05.

### Evaluation of the effects of apoptosis on the proliferation of *R. rickettsii* in BME26

BME26 cells were incubated with either Z-DEVD-Fmk (final concentration 10 μM), a caspase-3 inhibitor, or staurosporine (final concentration 400 nM). After 1 h, the cells were infected with *R. rickettsii*. The quantification of *R. rickettsii* in BME26 was determined after 6, 24, 48, 72, 96 and 120 h by qPCR, as described above. Three biological replicates for each treatment were analyzed. Differences in bacterial growth were determined by a Student’s t-test using GraphPad Prism software and considered to be significant when *P* < 0.05.

## Results

### Effects of rickettsial infection on the proteome of BME26 cells

Determination of the growth curve of *R. rickettsii* in BME26 cells revealed a doubling time of 8.5 h (Additional file 2: Figure S1a). Progressive infection of cells was observed over time, with the vast majority of the cells infected by 96 h after the beginning of infection (Additional file 2: Figure S1b–e). To determine changes in the proteome of BME26 cells in response to infection, we chose two time points on the *R. rickettsii* growth curve, one in the initial phase of the infection (6 h) and the other in the middle of the exponential phase of bacterial growth (48 h). Proteins extracted from noninfected (control) and *R. rickettsii-*infected BME26 cells were reduced, alkylated and digested with trypsin, and the resulting peptides were analyzed by LC–MS/MS. The raw data thus obtained were analyzed using the MaxQuant program against a database containing deduced amino acid sequences of transcripts of BME26 cells and protein sequences of bacteria of the genus *Rickettsia*.

A total of 1061 proteins were identified, among which 1056 correspond to tick proteins (Additional file 3: Table S1; Additional file 4: Table S2; Additional file 5: Table S3; Additional file 6: Table S4; Additional file 7: Table S5; Additional file 8: Table S6) and five to *Rickettsia* spp. proteins (Additional file 9: Table S7). Among the tick proteins, 789 were detected in all analyzed conditions (Additional file 10: Figure S2a). PCA of the quantitative protein features demonstrated a clear separation between the datasets of BME26 cells at 6 and 48 h post-infection (Additional file 10: Figure S2b). We observed separation between datasets of the control and infected cells at each of the two time points, although a slight overlap was observed at 48 h.

**Fig. 1:**
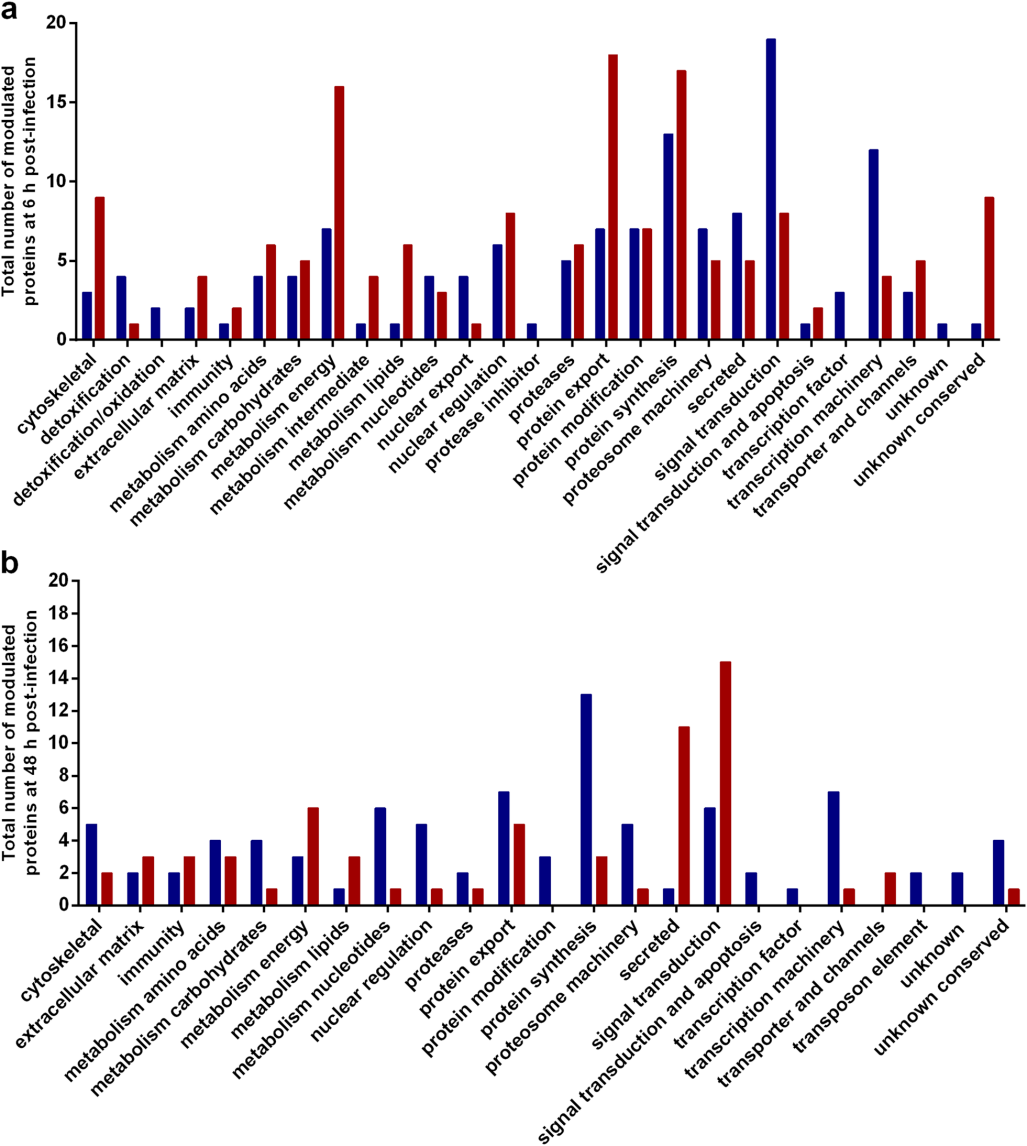
Functional classification of tick proteins modulated by *Rickettsia rickettsii* infection. Bars represent the total of proteins within each functional category that were upregulated (blue bars) or downregulated (red bars) at 6 (**a**) and 48 h (**b**) post-infection with *R. rickettsii*

At the time point of 6 h post-infection, 839 proteins common to both control and *R. rickettsii-*infected cells were identified (Additional file 10: Figure S2c). Among these, 47 presented a higher relative abundance (*P* < 0.05 and fold-change ≥ 1.5), and 92 exhibited a lower relative abundance (*P* < 0.05 and fold-change ≤ 0.67) in infected than in control cells (Additional file 3: Table S1). A total of 59 proteins were identified exclusively in the control cells (Additional file 4: Table S2) and 84 exclusively in *R. rickettsii*-infected cells (Additional file 5: Table S3) at 6 h post-infection. Thus, a total of 131 proteins were considered to be upregulated (i.e. proteins with higher relative abundance in infected cells or exclusively detected in this condition) and 151 to be downregulated (i.e. proteins with relative abundance lower in infected cells or identified exclusively in control cells) in the initial phase of infection. Regarding the time point of 48 h post-infection, we identified a total of 853 proteins that were common to both control and infected cells (Additional file 10: Figure S2d). Among these proteins, 19 showed a higher relative abundance and 24 presented a lower relative abundance in *R. rickettsii*-infected cells compared to noninfected cells (Additional file 6: Table S4). Thirty-nine proteins were identified exclusively in control cells (Additional file 7: Table S5), and 68 proteins were identified exclusively in infected cells (Additional file 8: Table S6). Thus, a total of 87 proteins were upregulated and 63 were downregulated at 48 h post-infection. The proteins modulated by infection were functionally classified (Fig. 1), as described by Karim and collaborators [31].

Regulatory components of the 20S and 26S subunits of the proteasome, which are included within the proteasome machinery category, were either upregulated (Table 1) or downregulated (Table 2) at 6 h post-infection. In addition, one proteasome inhibitor and one ubiquitin modifier were upregulated (Table 1), while one ATP-dependent Lon protease was downregulated at this same time point (Table 2). Despite a smaller number of proteins related to the proteasome machinery being modulated at 48 h post-infection, the majority were upregulated, including regulatory components of subunits 20S and 26S and one ubiquitin-protein ligase (Table 1). In contrast, the Skp1/Cullin/F-box containing complex (SCF) ubiquitin ligase component was downregulated (Table 2). Regarding the cytoskeletal and actin components, the subunits Arp2 and ArpC1/p41-Arc of the Arp2/3 complex were downregulated at 6 h (Table 2) while the ArpC5 subunit of the same complex and actin-binding proteins, including one spectrin, were upregulated (Table 1).

**Table 1:** Selected proteins upregulated at 6 or 48 h post-*Rickettsia rickettsii* infection of BME26 cells

Functional classi of proteins	6 h post-infection		48 h post-infection	
	ID	Annotation	ID	Annotation
ST/ APOPTOSIS	BME26USP-64008	Apoptotic chromatin condensation inducer in the nucleus	BME26USP-3827	Mucin-1—Negative regulation of intrinsic apoptotic signaling pathway in response to DNA damage by p53 class mediator
			BME26USP-77602	Fanconi anemia complementation group D2—negative regulation of apoptotic process
			BME26USP-73913	Cytokine-induced apoptosis inhibitor 1
PROT	BME26USP-3792	Small ubiquitin-related modifier 3	BME26USP-63775	26S Proteasome regulatory complex subunit RPN11
	BME26USP-1981	20S Proteasome regulatory subunit alpha type PSMA5/PUP2	BME26USP-4899	20S Proteasome regulatory subunit beta type PSMB1/PRE7
	BME26USP-61379	26S Proteasome regulatory complex subunit RPN12/PSMD8		
	BME26USP-61464	26S Proteasome regulatory complex ATPase RPT5	BME26USP-66020	Ubiquitin-protein ligase
	BME26USP-63407	proteasome inhibitor		
	BME26USP-67891	26S Proteasome regulatory complex subunit RPN7/PSMD6	BME26USP-68301	20S Proteasome regulatory subunit alpha type PSMA1/PRE5
	BME26USP-70300	26S Proteasome regulatory complex subunit RPN5/PSMD12		
CS	BME26USP-SigP-75480	Tyrosine protein kinase receptor tie-1	BME26USP-2996	Microtubule-associated protein partial
	BME26USP-71952	Actin-related protein arp2/3 complex subunit arpc5	BME26USP-5135	Tropomodulin and leiomodulin
	BME26USP-71823	Microtubule-binding protein	BME26USP-65063	Src substrate cortactin
	BME26USP-26177	Ca^2+^-Binding actin-bundling protein (spectrin) alpha chain	BME26USP-42758	Vinculin—Neuron projection morphogenesis/phagocytosis/actin/cytoskeleton/structural molecule/actin binding/cytoskeletal anchoring
			BME26USP-69366	Alpha tubulin

ST/Apoptosis, Signal transduction and apoptosis; PROT, proteasome machinery; CS, cytoskeletal functional classes, SRC

**Table 2:** Selected proteins downregulated at 6 or 48 h post-*R. rickettsii* infection of BME26 cells

Functional class of proteins	6 h post-infection		48 h post-infection	
	ID	Annotation	ID	Annotation
ST/ APOPTOSIS	BME26USP-3827	Mucin-1—Negative regulation of intrinsic apoptotic signaling pathway in response to DNA damage by p53 class mediator	–	–
	BME26USP-65523	Apoptosis inhibitor 5/fibroblast growth factor 2-interacting factor 2		
	BME26USP-74870	Clathrin assembly protein AP180		
	BME26USP-4568	Ubiquinone oxidoreductase b16.6 subunit/cell death		
PROT	BME26USP-65609	20S Proteasome regulatory subunit beta type PSMB4/PRE4	BME26USP-65272	SCF ubiquitin ligase Skp1 component
	BME26USP-64301	26S Proteasome regulatory complex ATPase RPT6		
	BME26USP-75557	20S Proteasome regulatory subunit alpha type PSMA2/PRE8		
	BME26USP-4899	20S Proteasome regulatory subunit beta type PSMB1/PRE7		
	BME26USP-77519	26S Proteasome regulatory complex subunit RPN10/PSMD4		
	BME26USP-23319	ATP-dependent Lon protease		
CS	BME26USP-75222	Alpha tubulin	BME26USP-26191	Protein tyrosine kinase
	BME26USP-71918	Actin-related protein Arp2/3 complex subunit Arp2		
	BME26USP-62031	Cdc42 homolog/positive regulation of protein kinase/maintenance of protein location/adherens junction		
	BME26USP-2688	GDP dissociation inhibitor/microtubule-associated complex/protein transport/Rab GDP-dissociation inhibitor	BME26USP-SigP-76633	Tyrosine kinase eph ephrin receptor family
	BME26USP-4897	Actin-related protein Arp2/3 complex subunit ARPC1/p41-ARC		
	BME26USP-25990	Dynein light intermediate chain		
	BME26USP-77773	Microtubule-actin cross-linking factor 1		

ST/apoptosis: signal transduction and apoptosis; PROT: proteasome machinery; CS: cytoskeletal functional classes, SCF, Skp1/Cullin/F-box containing complex

Among proteins within the signal transduction and apoptosis (ST/apoptosis) category, one apoptotic chromatin condensation inducer in the nucleus was upregulated at 6 h (Table 1). In contrast, mucin-1 (a negative regulator of the intrinsic pathway of apoptosis signaling in response to DNA damage by the p53 mediator) and the apoptosis inhibitor 5 (this one included in the immunity category) were downregulated (Table 2). Conversely, mucin-1 was upregulated 48 h post-infection (Table 1). Other factors that negatively regulate the apoptotic process (Fanconi anemia complementation group D2 and cytokine-induced apoptosis inhibitor 1) were also upregulated at 48 h (Table 1).

### Effects of *R. rickettsii* infection on the apoptotic process of BME26 cells

To evaluate if *R. rickettsii* exerted an effect on the apoptosis of BME26 cells, as suggested by the proteome data, the gDNA extracted from noninfected and infected cells was analyzed by electrophoresis in an agarose gel. Fragmentation of the gDNA was observed only in noninfected cells, starting at 96 h after the beginning of the experiment (Additional file 11: Figure S3). Subsequently, we assessed the activity of caspase-3 induced with staurosporine (a classic activator of apoptosis [32]) in BME26 cells pre-infected with *R. rickettsii* for 24, 36, and 48 h (Additional file 12: Figure S4). Caspase-3 activity was significantly lower in cells previously infected for 36 (*P* < 0.002) and 48 (*P* < 0.01) h when compared to noninfected cells.

**Fig. 2:**
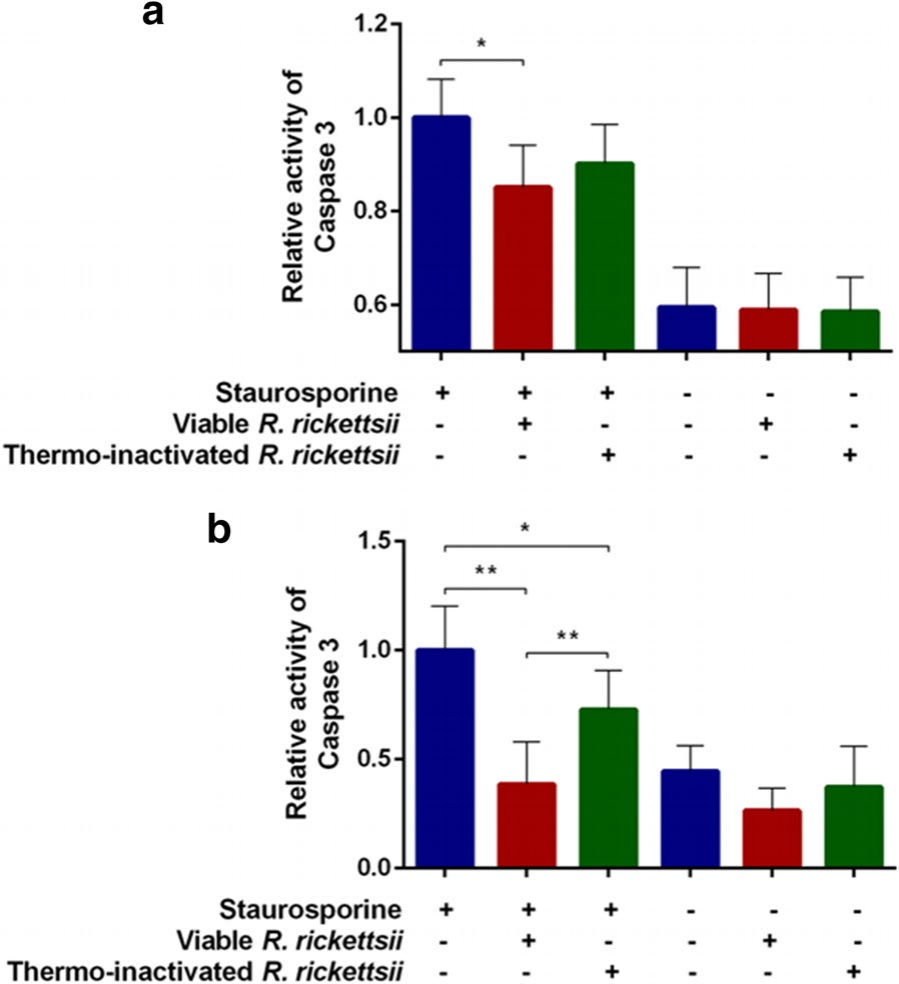
Caspase-3 activity in cells of the *Rhipicephalus microplus* (Canestrini) BME26 and *Amblyomma sculptum* (Berlese) embyronic cell lines (BME26 and IBU/ASE-16, respectively) exposed to either viable or thermo-inactivated *R. rickettsii*. The activity of caspase-3, induced or not by staurosporine, was evaluated in BME26 (**a**) and IBU/ASE-16 (**b**) cells exposed to viable (red bars) or thermo-inactivated (green bars) *R. rickettsii*. As a control, caspase-3 activity was determined in noninfected cells (blue bars). Forty-eight hours after exposure, the enzymatic activity was determined by measuring the release of the AMC fluorescent cleavage product from the synthetic fluorogenic substrate Ac-DEVD-AMC (emission excitation: 380–460 nm). The relative activity of caspase-3 (in units of arbitrary fluorescence [UAF]) represents the ratio of ΔUAF (UAF_60 min_ − UAF_0 min_) of each condition to the ΔUAF of noninfected cells treated with 400 nM staurosporine. Error bars: ± standard deviation of six measurements (*n* = 6). Asterisks indicate significant difference at *P < 0.05 and **p < 0.001 (Student’s t-test)

To assess if the inhibition of caspase-3 activity was a response of BME26 cells to components produced by live rickettsiae, cells were exposed to either viable or thermo-inactivated *R. rickettsii* (Fig. 2a). Only cells infected with viable bacteria had a significantly lower caspase-3 activity (*P* < 0.02) compared to the control. However, a slight—but not significant—reduction of caspase-3 activity was also observed between noninfected cells and cells exposed to thermo-inactivated rickettsiae. No difference was detected among the three groups in the absence of staurosporine. For comparison purposes, we conducted a similar experiment using an embryonic tick cell line of one primary vector of *R. rickettsii* in Brazil, *A. sculptum* (IBU/ASE-16) (Fig. 2b). The results showed that cells infected with live *R. rickettsii* presented a significantly lower caspase-3 activity (*P* < 0.0001) than the control. A significant lower caspase-3 activity was also detected in cells stimulated with thermo-inactivated bacteria than in noninfected cells (*P* < 0.007). On the other hand, caspase-3 activity was significantly higher in cells stimulated with thermo-inactivated bacteria than in cells infected with viable rickettsiae (*P* < 0.001). A lower caspase-3 activity was also detected in IBU/ASE-16 cells infected with viable bacteria than in noninfected cells without staurosporine induction, although the difference was not significant.

**Fig. 3:**
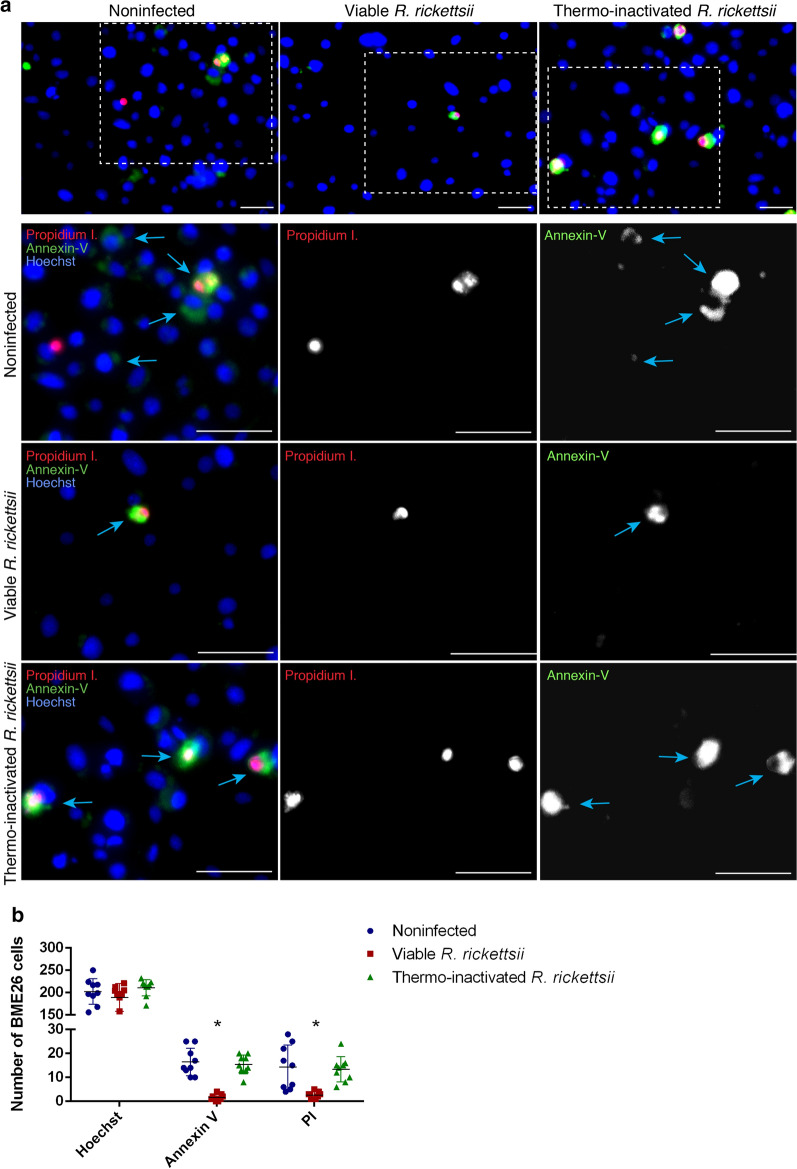
Phosphatidylserine externalization in BME26 cells exposed to either viable or thermo-inactivated *R. rickettsii*. Noninfected BME26 cells (control) or cells exposed for 48 h to either viable or thermo-inactivated *R. rickettsii* were treated with 400 nM staurosporine. After 6 h, cells were labeled with propidium iodide (red), annexin-V (green) and Hoechst (blue), and observed under a fluorescence microscope.** a** The panel at the top represents the three conditions at 40× magnification. The square areas outlined with broken lines are those areas presented in the images below the top panel, for better visualization. The light-blue arrows indicate annexin-V staining. Bar: 40 μm.** b** The graph represents the number of cells labeled with each probe in three independent images of each of the three biological replicates. Error bars: ± standard deviation. Asterisk indicates significant difference at *P < 0.001 (Student’s t-test)

The phosphatidylserine externalization in noninfected BME26 cells and cells exposed to viable or thermo-inactivated *R. rickettsii* was also assessed by live-cell fluorescence microscopy after labeling with annexin-V (Fig. 3a), which specifically binds to phosphatidylserine [33]. Cells were also labeled with the DNA probes Hoechst (staining live cells) and PI (staining dead/apoptotic cells). The number of annexin-V-labeled cells was significantly lower in cells infected with viable *R. rickettsii* than in noninfected cells (*P* <0.0001) or cells stimulated with thermo-inactivated rickettsiae (*P* <0.0001) (Fig. 3b). The number of dead cells (those labeled with PI) was also significantly lower among cells that were infected with viable bacteria than in the other two groups (*P* < 0.0002).

### Effects of caspase-3 activation on the proliferation of *R. rickettsii* in BME26 cells

**Fig. 4:**
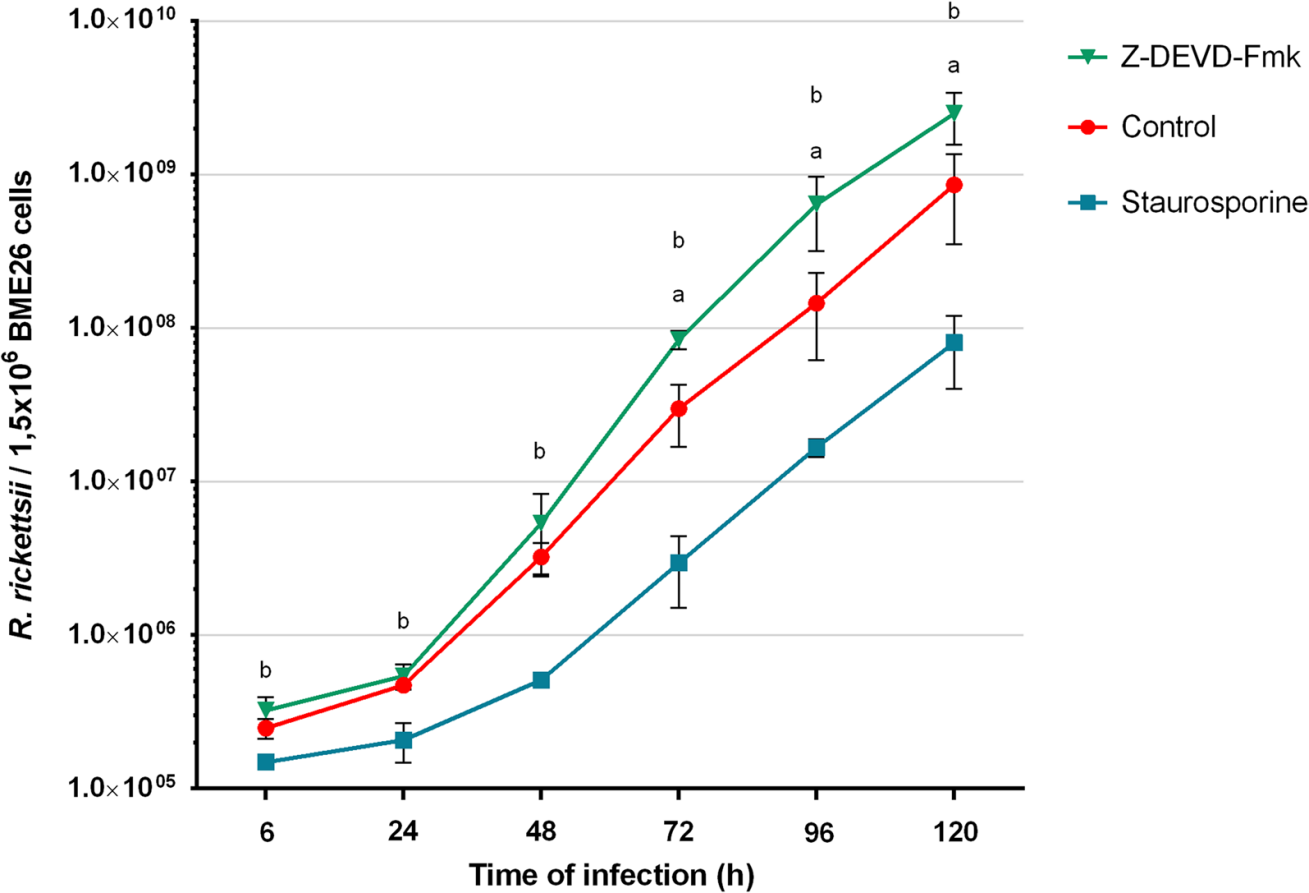
Growth curve of *R. rickettsii* in BME26 cells treated with either staurosporine or the caspase-3 inhibitor Z-DEVD-Fmk. BME26 cells were treated with staurosporine (400 nM) or the caspase-3 inhibitor Z-DEVD-Fmk (10 μM). Untreated cells served as the control. After 1 h, cells were inoculated with *R. rickettsii*. The number of rickettsiae after 6, 24, 48, 72, 96 and 120 h was determined by quantitative PCR using specific primers and a hydrolysis probe for the single-copy gene *gltA* of *Rickettsia* spp. ^a^Significant difference in rickettsial growth in cells treated with Z-DEVD-Fmk (*P* < 0.02, Student’s t-test),^b^ significant difference in rickettsial growth in cells treated with staurosporine (*P* < 0.007, Student’s t-test), compared to the control. Error bars: ± standard deviation (*n* = 3)

To assess the effect of the activation of caspase-3 on the proliferation of *R. rickettsii*, the bacterial growth in BME26 cells treated with either staurosporine or Z-DEVD-Fmk, a caspase-3 inhibitor, was evaluated (Fig. 4). *Rickettsia rickettsii* growth was significantly higher in BME26 cells treated with Z-DEVD-Fmk than in untreated cells (control) from 72 to 120 h post-infection (*P* < 0.02). Specifically, the doubling time of the bacterial population in untreated cells was 7.7 h, compared to 6.2 h in cells treated with Z-DEVD-Fmk. On the other hand, the induction of caspase-3 activity with staurosporine negatively impacted bacterial growth, causing an increase in the doubling time to 11.3 h. In this condition, a significant difference in bacterial growth compared to the control (*P* < 0.007) was detected at all time points, starting at the 6 h post-infection.

## Discussion

Although arthropods lack the adaptive response of vertebrates, a series of effective cellular and humoral reactions are triggered after a microbial invasion to control infection [34]. Different types of cell death, including apoptosis, also play an important role in the immune defense of arthropods (as well as of other animals), as the infected cells are eliminated, thereby preventing the dissemination of the infectious agent [35–38]. To counteract the host response, the microorganisms use different strategies, including the inhibition of apoptosis, to ensure their survival and proliferation [35, 39]. For example, Ats-1, an effector of the type IV secretion system (T4SS) of the tick-borne bacterium *Anaplasma phagocytophilum* (causative agent of human granulocytic anaplasmosis), inhibits the mitochondrial release of the pro-apoptotic cytochrome *c*, delaying host cell apoptosis [40]. The delay of apoptosis is crucial for the completion of the bacterial life-cycle within neutrophils [41, 42]. *Anaplasma phagocytophilum* is also capable of manipulating the apoptosis of its tick vector [43–47].

In the present study, we showed that the infection with *R. rickettsii* alters the proteome of BME26 cells in a time-dependent manner. Regarding proteins involved in apoptosis, certain negative regulators were downregulated at 6 h post-infection with *R. rickettsii* but upregulated at 48 h post-infection. As mentioned earlier in this article, previous studies have shown that *R. rickettsii* is capable of inhibiting apoptosis in human endothelial cells [14–16]. In this process, the activation of the nuclear transcription factor-kappa B (NF-kB) by infection is crucial [14]. NF-kB controls the levels and/or the localization of proteins from the B-cell lymphoma 2 (Bcl-2) family [15], which, in turn, prevents the activation of the apical caspases-8 and -9, as well as the effector caspase-3 [16]. We therefore hypothesized that *R. rickettsii* could also subvert apoptosis in tick cells, as suggested by proteome data. The fragmentation of DNA, a classic marker of apoptotic cells [48], was detected only in noninfected BME26 cells. Moreover, the activity of the executioner caspase-3 was assessed in two distinct tick cell lines, the BME26 from *R. microplus* and the IBU/ASE-16 from *A. sculptum*. The activity of caspase-3 was significantly lower in both *R. rickettsii*-infected cell lines in comparison to noninfected cells. Caspase-3 was also significantly lower in IBU/ASE-16 cells exposed to thermo-inactivated rickettsiae than in the control cells, suggesting that components produced by live *R. rickettsii* may inhibit caspase-3 activity. Inhibition of caspase-3 activity by thermo-inactivated bacteria compared to the control might be due to the leakage components of dead bacteria, as BME26 cells exhibit a phagocytic activity [8].

The translocation of phosphatidylserine from the inner to the outer layer of the plasma membrane is another important feature of apoptotic cells, allowing their recognition and elimination by phagocytic cells [49]. A higher number of noninfected BME26 cells were labeled with annexin-V when compared to the cells infected with viable *R. rickettsii*, showing that infection reduces the exposure of phosphatidylserine. Calreticulin is considered to be the second recognition ligand of the apoptotic cell, binding to the low-density lipoprotein (LDL)-receptor-related protein of the phagocytic cell [50]. Interestingly, proteome data showed that one LDL-receptor-related protein was upregulated at 6 h (Additional file 3: Table S1) and downregulated at 48 h (Additional file 6: Table S4) post-infection of BME26 cells.

The proteome of BME26 cells also revealed that components of the ubiquitin–proteasome system were modulated at both time points after infection of BME26 cells. This system plays a key role in the degradation of damaged endogenous proteins or those with errors of synthesis, as well as in the regulation of several cellular processes [51, 52], including apoptosis [53]. The target proteins are marked for degradation by ubiquitination, which is catalyzed by three enzymes: ubiquitin-activating enzyme (E1), ubiquitin-conjugating enzyme (E2) and ubiquitin-protein ligase (E3) [54, 55]. Interestingly, it was previously reported that the chemical inhibition of the proteasome system of human endothelial cells by MG-132 leads to rapid death upon infection with *R. rickettsii*, as it prevents the degradation of the NF-kB inhibitor and, consequently, the apoptotic process is not inhibited [14]. In our study, at 6 h post-infection with *R. rickettsii*, the ubiquitin C-terminal hydrolase, an enzyme that cleaves ubiquitin from proteins and other molecules [54], was downregulated. Moreover, a proteasome inhibitor was upregulated, suggesting that the ubiquitin–proteasome system is inhibited at the beginning of the infection. The modulation of the proteasome inhibitor was not detected at 48 h post-infection. At 48 h, one ubiquitin ligase (E3) was also upregulated. In* Ixodes scapularis*, the knockdown of the X-linked inhibitor of apoptosis protein (XIAP), which possesses a really interesting new gene (RING) zinc-finger motif that exhibits E3-ubiquitin ligase activity, increased the colonization by* Anaplasma phagocytophilum*, showing that E3 is important for the control of infection [56]. Indeed, it has been described that ubiquitination regulates arthropod immune responses against microorganisms [55].

In our study, proteins of the cytoskeletal category were also modulated by infection of BME26 cells with *R. rickettsii*. Among these, we highlight subunits of the Arp2/3 complex and spectrin. Members of the genus *Rickettsia*, which compose the spotted fever group, polymerize the host actin to move within the host cell as well as to invade adjacent cells [57–60]. This process is mediated by the bacterial RickA protein, which activates the Arp2/3 complex of the host cell in one of the poles of the bacterium, triggering actin polymerization and the propulsion of the bacterium. Therefore, the modulation of proteins of the Arp2/3 complex during infection is important to assure the spread of rickettsiae. More recently, actin-related proteins have also been associated with apoptosis [61, 62]. Indeed, it was previously reported that spectrin is involved in rearrangement of the cell cytoskeletal in the case of infection [63], which can also activate apoptosis [64]. Interestingly, the knockdown of spectrin caused a diminution of *A. phagocytophilum* load in the midgut, salivary glands and the embryonic cell line ISE6 of *I. scapularis* [43].

Importantly, the growth of *R. rickettsii* was higher in BME26 cells treated with the caspase-3 inhibitor and lower in those treated with staurosporine, indicating that the activation of apoptosis is detrimental while its inhibition favors rickettsial proliferation. Moreover, it is also possible that *R. rickettsii* cannot efficiently invade apoptotic cells, which further reinforces the importance of regulating this process to guarantee cellular invasion.

## Conclusions

Taken together, our data showed that *R. rickettsii* modulate the proteome of BME26 cells, including proteins enrolled with apoptosis, and exerts an inhibitory effect on the apoptosis in tick cells, which seems to be advantageous to ensure its proliferation in tick cells.

## Supplementary information

**Additional file 1:** FASTA file. Deduced amino acid sequences from transcripts of BME26 cells and protein sequences from bacteria of the genus *Rickettsia*.

**Additional file 2: Figure S1.** Growth curve and photomicrograph of *R. rickettsii* in BME26 cells. The number of rickettsiae was determined at each time point by qPCR using specific primers and a hydrolysis probe for the single-copy gene *gltA* (**A**). Error bars: ± SD (*n* = 3). Aliquots of BME26 were removed at 24 h (**B**), 48 h (**C**), 72 h (**D**) and 96 h (**E**) post-infection, stained by Gimenez and visualized under a light microscope. A high concentration of rickettsiae (colored in purple and indicated by the arrows) was observed in the nuclei (*n*) of the cells at 24 (**b**) and 48 h (**c**).

**Additional file 3: Table S1.** Proteins differentially expressed in BME26 cells after 6 h of infection with *R. rickettsii*.

**Additional file 4: Table S2.** Proteins exclusively identified in noninfected BME26 cells (control) at 6 h.

**Additional file 5: Table S3.** Proteins exclusively identified in *R. rickettsii*-infected BME26 cells at 6 h.

**Additional file 6: Table S4.** Proteins differentially expressed in BME26 cells after 48 h of infection with *R. rickettsii*.

**Additional file 7: Table S5.** Proteins exclusively identified in noninfected BME26 cells (control) at 48 h.

**Additional file 8: Table S6.** Proteins exclusively identified in *R. rickettsii*-infected BME26 cells at 48 h.

**Additional file 9: Table S7.***Rickettsia* spp. proteins identified in BME26 cells by LC-MS/MS.

**Additional file 10: Figure S2.** Global sample characterization and comparison of proteins identified in noninfected and *R. rickettsii*-infected BME26 cells by LC-MS/MS. Venn diagrams displaying the total number of tick proteins that were exclusively detected in one condition or shared among different conditions:** A** noninfected (control) and *R. rickettsii*-infected cells at 6 or 48 h post-infection.** B** Principal component analysis (PCA) plot of protein datasets (*C* control biological replicates,* I* infected biological replicates, 6 or 48 h post-infection;* PC1* principal component 1,* PC2* principal component 2).** C**,** D** Venn diagrams showing proteins that were exclusively detected or were detected in both noninfected and *R. rickettsii* infected cells at 6 h (**C**) and 48 h (**D**) post-infection. Among shared proteins, only those with *P* < 0.05 and a fold-change ≥ 1.5 or ≤ 0.67 were considered modulated by infection.

**Additional file 11: Figure S3.** Analysis of the gDNA extracted from noninfected and *R. rickettsii*-infected BME26 cells by agarose gel electrophoresis. The gDNA extracted from noninfected (control) or *R. rickettsii*-infected BME26 cells at 6, 24, 48, 72, 96 and 120 h post-infection was separated on a 1% agarose gel electrophoresis stained with RED™ Gel and visualized under UV light. DNA marker size (bp) is shown.

**Additional file 12: Figure S4.** Caspase-3 activity in BME26 at different times after infection with *R. rickettsii*. BME26 cells previously infected with *R. rickettsii* for 24, 36, and 48 h (red bars) were treated with 400 nM staurosporine. As a control, noninfected cells (blue bars) were also treated with 400 nM of staurosporine. The enzymatic reaction was carried out at 37 °C for 60 min, and hydrolysis was measured by the release of the fluorescent cleavage product AMC from the synthetic fluorogenic substrate Ac-DEVD-AMC (emission–excitation: 380–460 nm). The relative activity of caspase-3 (in units of arbitrary fluorescence [UAF]) represents the ratio of ΔUAF (UAF_60 min_ − UAF_0 min_) of each condition to the ΔUAF of noninfected cells in each time analyzed. Error bars: ± SD (*n* = 3). *Significantly different at *P* <0.05 (Student’s t-test).

## Data Availability

The mass spectrometry raw files were submitted to PRIDE (https://www.ebi.ac.uk/pride/) with the submission number PXD017942. The BME26 RNA-seq raw data were deposited to the Sequence Read Archives (SRA) of the NCBI under bioproject number PRJNA607772 and the Transcriptome Shotgun Assembly (TSA) project has been deposited at DDBJ/EMBL/GenBank under the accession GINU00000000 (the version described here is the first version, GINU01000000). The authors declare that all other data supporting the findings of this study are available within the article and its Additional Information files.
