# Supplementary figures and images for: The intracellular bacterium Rickettsia rickettsii exerts an inhibitory effect on the apoptosis of tick cells
Source: Parasit Vectors. 2020 Dec 1;13:603. doi: 10.1186/s13071-020-04477-5 (PMC7706286; doi:10.1186/s13071-020-04477-5)

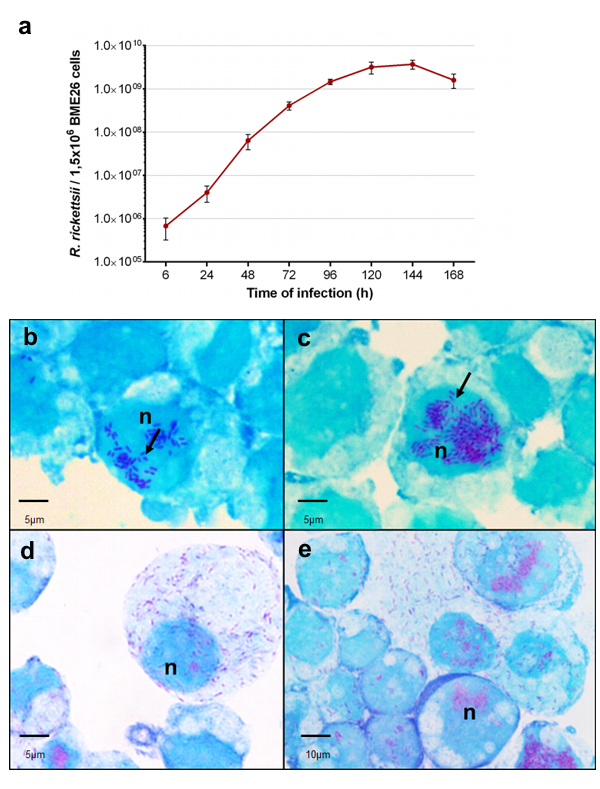

Supplement: Supplementary file 2 — Additional file 2: Figure S1. Growth curve and photomicrograph of R. rickettsii in BME26 cells. The number of rickettsiae was determined at each time point by qPCR using specific primers and a hydrolysis probe for the single-copy gene gltA (A). Error bars: ± SD (n = 3). Aliquots of BME26 were removed at 24 h (B), 48 h (C), 72 h (D) and 96 h (E) post-infection, stained by Gimenez and visualized under a light microscope. A high concentration of rickettsiae (colored in purple and indicated by the arrows) was observed in the nuclei (n) of the cells at 24 (b) and 48 h (c). [file 13071_2020_4477_MOESM2_ESM.tif]

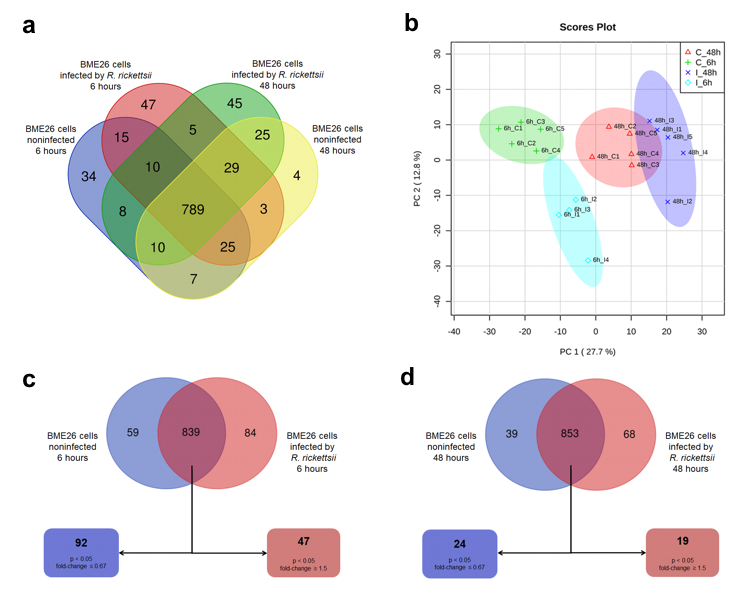

Supplement: Supplementary file 10 — Additional file 10: Figure S2. Global sample characterization and comparison of proteins identified in noninfected and R. rickettsii-infected BME26 cells by LC-MS/MS. Venn diagrams displaying the total number of tick proteins that were exclusively detected in one condition or shared among different conditions: A noninfected (control) and R. rickettsii-infected cells at 6 or 48 h post-infection. B Principal component analysis (PCA) plot of protein datasets (C control biological replicates, I infected biological replicates, 6 or 48 h post-infection; PC1 principal component 1, PC2 principal component 2). C, D Venn diagrams showing proteins that were exclusively detected or were detected in both noninfected and R. rickettsii infected cells at 6 h (C) and 48 h (D) post-infection. Among shared proteins, only those with P < 0.05 and a fold-change ≥ 1.5 or ≤ 0.67 were considered modulated by infection. [file 13071_2020_4477_MOESM10_ESM.tif]

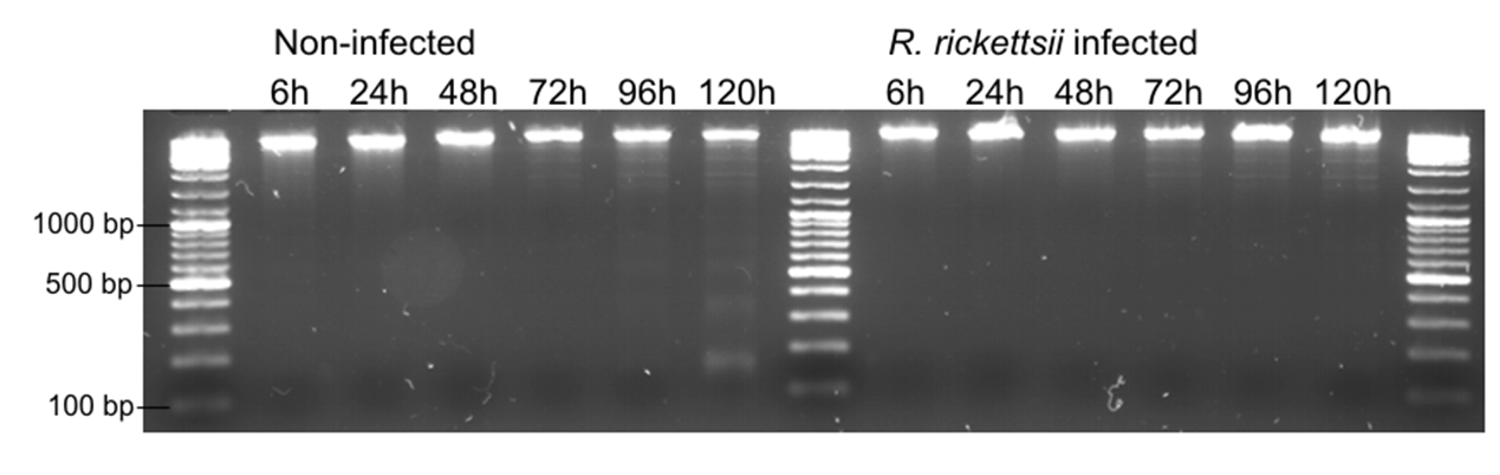

Supplement: Supplementary file 11 — Additional file 11: Figure S3. Analysis of the gDNA extracted from noninfected and R. rickettsii-infected BME26 cells by agarose gel electrophoresis. The gDNA extracted from noninfected (control) or R. rickettsii-infected BME26 cells at 6, 24, 48, 72, 96 and 120 h post-infection was separated on a 1% agarose gel electrophoresis stained with RED™ Gel and visualized under UV light. DNA marker size (bp) is shown. [file 13071_2020_4477_MOESM11_ESM.tif]
